# Unraveling Root Development Through Single-Cell Omics and Reconstruction of Gene Regulatory Networks

**DOI:** 10.3389/fpls.2021.661361

**Published:** 2021-05-04

**Authors:** Laura Serrano-Ron, Javier Cabrera, Pablo Perez-Garcia, Miguel A. Moreno-Risueno

**Affiliations:** Centro de Biotecnología y Genómica de Plantas (Universidad Politécnica de Madrid–Instituto Nacional de Investigación y Tecnología Agraria y Alimentaria), Campus de Montegancedo, Pozuelo de Alarcón, Madrid, Spain

**Keywords:** single-cell RNA-seq, gene regulatory networks, root development, organogenesis, cell fate

## Abstract

Over the last decades, research on postembryonic root development has been facilitated by “omics” technologies. Among these technologies, microarrays first, and RNA sequencing (RNA-seq) later, have provided transcriptional information on the underlying molecular processes establishing the basis of System Biology studies in roots. Cell fate specification and development have been widely studied in the primary root, which involved the identification of many cell type transcriptomes and the reconstruction of gene regulatory networks (GRN). The study of lateral root (LR) development has not been an exception. However, the molecular mechanisms regulating cell fate specification during LR formation remain largely unexplored. Recently, single-cell RNA-seq (scRNA-seq) studies have addressed the specification of tissues from stem cells in the primary root. scRNA-seq studies are anticipated to be a useful approach to decipher cell fate specification and patterning during LR formation. In this review, we address the different scRNA-seq strategies used both in plants and animals and how we could take advantage of scRNA-seq to unravel new regulatory mechanisms and reconstruct GRN. In addition, we discuss how to integrate scRNA-seq results with previous RNA-seq datasets and GRN. We also address relevant findings obtained through single-cell based studies and how LR developmental studies could be facilitated by scRNA-seq approaches and subsequent GRN inference. The use of single-cell approaches to investigate LR formation could help to decipher fundamental biological mechanisms such as cell memory, synchronization, polarization, or pluripotency.

## Introduction

Cells are the units of all biological systems. However, the functionality of cells in multicellular organisms requires their specification into tissues and cell types, and thus cells acquire different identities. It is anticipated that the analysis of multicellular organisms at the single-cell level will greatly facilitate the understanding of the mechanisms that govern specific biological processes (Macosko et al., 2015; Ziegenhain et al., 2017).

Cell identity can be understood as the integration of factors such as morphology, phenotype and function (which are related to the present), lineage (related to the past), and molecular state (which determines the future) (Morris, 2019). Usually, cell types are classified by features such as morphology, location, and molecular profile. The recent development of single-cell omics methods comes as a useful approach to discern cell types based on their molecular fingerprints. Furthermore, the use of these methods have facilitated the ability to gain new insights and obtain results that were thought to be unattainable a few years ago such as the generation of a cell atlas of the whole planarian (Plass et al., 2018), the discovery of new types of human blood cells (Villani et al., 2017), or unraveling neuron programming from embryonic stem cells (Velasco et al., 2017). In this review, we summarize single-cell RNA-sequencing (scRNA-seq) strategies as well as the use of these datasets to reconstruct predictive Gene Regulatory Networks (GRN). In addition, we discuss the integration of scRNA-seq results with already available RNA-seq datasets and GRN. We also review recent advances eased by these technologies in various organisms. Finally, we propose that scRNA-seq approaches can facilitate the identification of unknown regulatory mechanism during lateral root formation and propose possible single-cell omics experiments that can address remaining biological questions in the field.

## Single-Cell Omics Approaches

Single-cell omics technologies allow us to study multicellular organisms in an unbiased manner. As each cell is analyzed separately from the rest, specific molecular marks can be used to associate cells with existing molecular patterns, thus defining cell populations without previous assumptions. In contrast, approaches based on biomarkers or microdissection assign cells to predefined populations, which can potentially cause inaccurate results by mixing different types of cells. Single-cell omics technologies use different isolation methods and various types of data can be obtained: transcriptomic, proteomic, metabolomic, epigenetic data, and others.

### Isolation of Cells

An initial isolation step is required in any type of single-cell experiment. This has been specially challenging in plants as the cell wall prevents cell separation. Plant cells can be physically isolated through micromanipulators and micropipettes, or through laser microdissection. While these methods can be used in single-cell experiments, their low throughput and experimental difficulty have reduced their use; although these methods are considered to be precise and a labeling step could not be required (Thakare et al., 2014; Anjam et al., 2016; Zeb et al., 2019). For single-cell omics analyses, the plant cell wall is normally enzymatically digested allowing cell disaggregation to generate protoplasts (Birnbaum et al., 2005). As protoplasting facilitates high throughput processing in subsequent single-cell isolation methods, it has become one of the preferred techniques to disaggregate plant cells (Prakadan et al., 2017; Mincarelli et al., 2018). Protoplasting can generate a stress response in cells, thereby it can potentially alter their transcriptomes. However, it has been shown that changes in gene expression induced by the protoplasting procedure are reduced. Moreover, genes induced by protoplasting have been identified, so they can be easily ruled out from subsequent analyses (Birnbaum, 2003; Villarino et al., 2016).

As an alternative to protoplasting, nuclei isolation has been used in single cell experiments. Nuclei isolation has become the preferred isolation technique in animals for single-cell transposase-accessible chromatin sequencing (scATAC-seq) and SCI- seq. In scATAC-seq library adaptors are inserted into open chromatin regions to determine chromatin accessibility, while in SCI- seq, nucleosomes bound to genomic DNA are removed to generate uniformly distributed sequence reads followed by an assessment of copy-number variants (Vitak et al., 2017). Nuclei isolation for single-cell experiments can be achieved by enzymatic digestion of the cell membrane and subsequent centrifugation (Habib et al., 2016). The main advantages of single-nucleus- over single-cell isolation in single-cell experiments are the higher representation of rare cell types and the apparently lack of induced stress response genes (Wu et al., 2019). Nuclei isolation for single-cell experiments in plants is in the process of being implemented, while previously microarray and RNA-seq of plant nuclei were successfully performed using Isolation of Nuclei in Tagged Cell Types (INTACT) (Deal and Henikoff, 2010; Reynoso et al., 2018). In this method, nuclei of the desired cell type are labeled through the transgenic expression of a tagged protein, which can be later used for affinity purification. INTACT could be used in plant single-cell experiments as an alternative to protoplasting.

**FIGURE 1:**
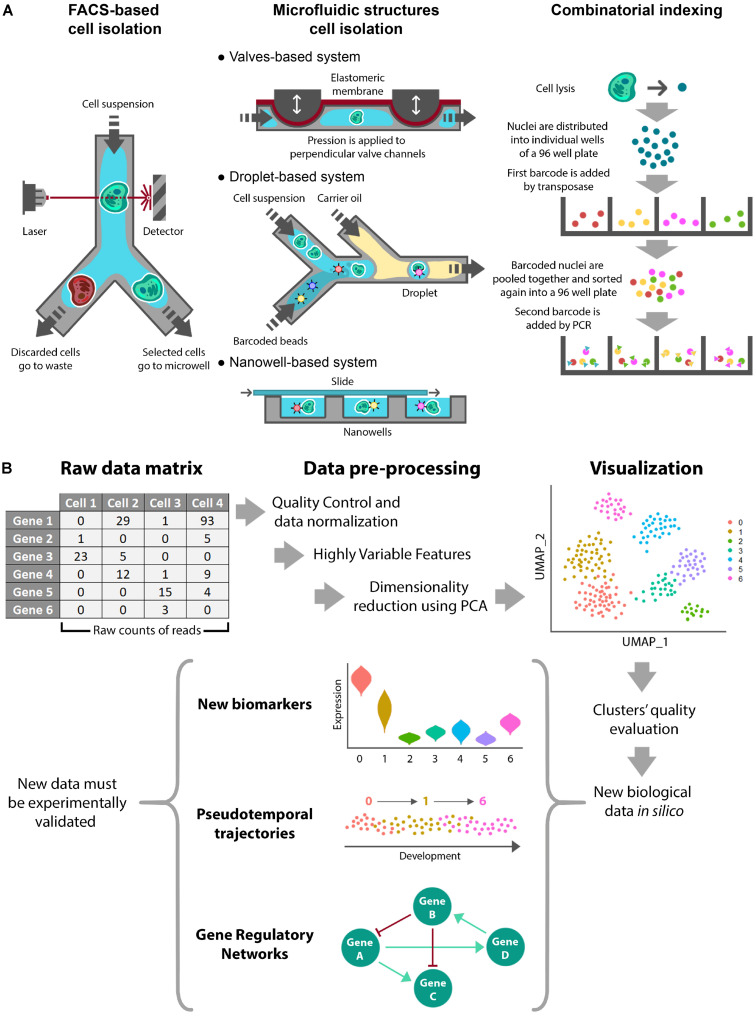
Single-cell Omics experimental procedures. Schematic representation of a single-cell omics experiment showing **(A)** the different available methods for cell isolation and **(B)** a standardized workflow for *in silico* processing of RNA sequencing data.

Once cells or nuclei are disaggregated, the main isolation methods prior to single-cell experiments are the following (Figure 1A):

• **FACS-Based Cell Isolation.** Fluorescence-Activated Cell Sorting (FACS) is a well-known method that utilizes flow cytometry to profile fluorescently marked cells. After fluorescence detection, individual cells are sorted and deposited into microtiter plates (Ramsköld et al., 2012; Jaitin et al., 2014). This approach is broadly used as it is compatible with different workflows and has the ability to automatically select the desired cells based on fluorescence and other cell characteristics. The main drawbacks concern cell damage, the large amount of initial material and the cost (Zeb et al., 2019).
• **Microfluidic Structures Cell Isolation.** These approaches are based on microfluidic devices, which typically are valves, droplets, and nanowells (Prakadan et al., 2017). Valves-based systems rely on microchannels made of an elastic membrane that can be deflected by applying pressure to block the flow and confine individual cells (Hong and Quake, 2003). Droplet-based systems make use of aqueous droplets in inert carrier oil. Individual cells are captured in droplets because they are loaded at low densities to obtain, at most, a single element per drop. In addition, one barcoded bead and lysis buffer are included in each droplet (Klein et al., 2015; Macosko et al., 2015). Finally, nanowell-based methods use cells at low concentration to encapsulate individual cells. In this case, roofless nanolitre-scale wells are filled with the cell suspension by gravity and then sealed on the top with a slide (Gierahn et al., 2017; Prakadan et al., 2017). In comparison with the FACS/plates-based method, these approaches can reduce the reagent cost per cell and maximize throughput due to the small size of the microfluidic devices. As cell isolation and DNA amplification are integrated in these methods, they are time and cost effective. In contrast, the main disadvantages of these methods are higher rates of cell damage and lower purity of the selected cells (Prakadan et al., 2017; Zeb et al., 2019). In occasions, these methods have been associated to lower depth of sequencing.
• **Combinatorial Indexing.** These methods are used to label and classify isolated nuclei. SCI-seq was the first single-cell whole-genome sequencing method using a combinatorial indexing strategy (Vitak et al., 2017). Combinatorial indexing normally uses a two-step barcoding workflow to label cell nuclei and DNA molecules. First, nuclei are isolated in several small pools, each one receiving a primary transposase-based barcode. After adding the first barcode, nuclei are mixed together and sorted again into small pools, when a second barcode is added by PCR to each pool. This way, each nucleus receives a unique combination of barcodes that identifies it (Vitak et al., 2017; Mincarelli et al., 2018). This method comes as an alternative to physical compartmentalization, eliminating the requirement for custom equipment. An additional advantage is its high throughput. On the contrary, shallowness of subsequent sequencing can be mentioned as its main drawback (Mincarelli et al., 2018).

### Molecular Profiling

The available single-cell methods enable the measurement of a catalog of cell parameters. Most single-cell approaches have addressed the identity of the cell (Stuart and Satija, 2019), which included the analysis of particular aspects of the transcriptome (Picelli et al., 2013; Macosko et al., 2015), genome (Navin et al., 2011; Vitak et al., 2017), epigenome (Gomez et al., 2013; Buenrostro et al., 2015; Lake et al., 2018), and proteome (Darmanis et al., 2016; Stoeckius et al., 2017). The specific methods available for each one of these modalities are reviewed in Stuart and Satija (2019).

More recently, efforts have focused on simultaneously analyzing several of the transcriptome, genome, epigenome, or proteome parameters for each single cell. This is known as multimodal profiling and anticipates a more profound understanding of the biology of the cell. Examples of these types of analyses are scG&T-seq (simultaneous measurement of genomic DNA and mRNA) and scM&T-seq (simultaneous measurement of DNA methylation and mRNA). Other cases of multimodal profiling are the cell lineage tracing methods scGESTALT, ScarTrace, and LINNAEUS. These methods infer lineage relationships between groups of cells based on shared DNA mutations, simultaneously analyzing the clonal history of the cell and its transcriptomic identity (Macaulay et al., 2015; Angermueller et al., 2016; Alemany et al., 2018; Raj et al., 2018; Spanjaard et al., 2018; Stuart and Satija, 2019).

### Data Processing and *in silico* Analysis

Once data are obtained and quantified, they are arranged in a matrix containing the extracted biological features per cell (Figure 1B). As the most commonly used analysis is scRNA-seq, we will focus on this type of data. scRNA-seq data are presented as a digital gene expression matrix of read counts per gene (in rows) and per cell (in columns). Many studies analyze these data using Seurat, which is used as an R package. Seurat aims to dissect heterogeneity from single-cell transcriptomic measurements integrating diverse types of single-cell data. The specific data processing workflow is comprehensively explained at the command level in the Seurat developers’ website^1^ (Butler et al., 2018; Stuart et al., 2019). The workflow involves the following steps (Figure 1B):

• **Quality Control and Normalization.** This first step selects the cells that will be used for subsequent analyses. This is performed through different quality control filters. Although Seurat pipeline is originally designed for animal tissues, similar quality controls can be used in plants such as the number of unique genes or molecules per cell and/or the percentage of reads that map to the mitochondrial genome (mitochondrial reads are expected to remain constant). Typical desired values for a cell are between 200 and 2,500 unique feature counts/cell and between 1 and 5% of mitochondrial counts/cell. In addition, quality controls in plants can be extended to chloroplast/plastid-derived counts (Shulse et al., 2019), which are expected to remain constant in an organ- or tissue- dependent manner. Next, selected cells are processed in order to normalize counts through different algorithms. Several of these algorithms involve regression analysis and removal of unwanted sources of variation.
• **Identification of Highly Variable Features.** Most variable features, i.e., genes with most different expression values among the normalized dataset, are used to perform dimensionality reduction and clustering. The statistical methods that can be used for normalization in Seurat are the natural logarithmic or centered logarithmic transformation of the count ratio and the scaled non-logarithmic transformation of the count ratio. To select the top variable features, Seurat assigns a dispersion value for each gene. This dispersion value can be the standard deviation, the expected variance fitted by a polynomial regression or the *z*-score. Finally, the genes with the highest dispersion values are selected. In addition, several statistical methods have been developed to obtain the differentially expressed genes from scRNA-seq experiments. The majority of these algorithms (SCDE, MAST, SigEMD, DEsingle, SINCERA, DESeq2, edgeR) are implemented for R and (D3E) for python (Wang et al., 2019; Hoffman et al., 2020).
• **Linear Dimensional Reduction and Determination of the Dimensionality of the Dataset.** After scaling the data (linear transformation), a principal component analysis (PCA) is performed using the most variable features previously determined. The primary sources of heterogeneity in the dataset (genes and cells) can then be explored using various methods. This information helps to assess the number of principal components that should be considered to accurately represent the dataset.
• **Clustering and Visualization.** Cells are clustered using the selected principal components of the PCA. As 5–10 principal components are normally used for clustering in Seurat, the resulting clusters cannot be easily represented by PCA plotting, so they are normally visualized by non-linear dimensional reduction methods, such as t-distributed Stochastic Neighborhood Embedding (tSNE) or Uniform Manifold Approximation and Projection (UMAP) (Maaten and Hinton, 2008; McInnes et al., 2018). These methods preserve local similarities while they represent data/cells in a non-linear way that better captures clustering as compared with PCA plotting. Next, differentially expressed genes among clusters can be identified. These genes have enriched expression in specific clusters and represent biomarkers. The following step usually consists of assigning specific cell type identities to the clusters. To do so, a typical approach is examining the expression of known cell type markers (Denyer et al., 2019; Ryu et al., 2019). A complementary option to identify cell types or assign identity to clusters is through the Index of Cell Identity (ICI) method (Efroni et al., 2015). The ICI method computes a score for each cell based on libraries of gene expression profiles for known cell types. The resulting score gives the relative contribution of each known cell type to the identity of the cell, thus facilitating its identification. An additional advantage of the ICI method is that cells with mixed identities can be categorized.

### Pseudotemporal and Network Analyses

scRNA-seq data can be used to reconstruct GRN as well as to perform the so called pseudotime analyses. Pseudotime studies aim to order cells along a one-dimensional axis that represents a continuous process such as differentiation or development. These methods assign a relative time to the cells to compute their order. Even though development or differentiation processes imply differences in gene expression profiles, progression can occur at different speeds depending on each cell. Thus, cell transcriptomics are analyzed as state-dependent instead of as time-dependent features (Rich-Griffin et al., 2020). Most commonly used methods include Monocle (Trapnell et al., 2014), Wishbone (Setty et al., 2016), Diffusion (Haghverdi et al., 2016), and Velocyto (La Manno et al., 2018). In addition, as Velocyto is based on the measurement of intronic RNA reads (defined as RNA velocity), it can infer the future transcriptional state of cells. This addresses some of the problems found in the other methods such as rooting and branching of the trajectories (La Manno et al., 2018; Stuart et al., 2019). However, the lower abundance of intronic reads detected in plants can hinder the annotation of gene splicing rates, thus potentially rendering less reliable results for Velocyto in plants (Li et al., 2016; Jean-Baptiste et al., 2019).

Development of microarray and RNA-seq technologies have greatly contributed to the generation of a large amount of expression data, facilitating the identification of molecular mechanisms regulating cell-type-specific gene expression during development or stress (Brady et al., 2007; Dinneny et al., 2008). In parallel, bioinformatics methods were developed to infer genetic interactions using sequenced transcriptomes, thus making GRN reconstruction possible. GRN represent gene regulatory dependencies which are mathematically inferred from transcriptomic data. In GRN, the nodes represent the genes, and the edges the positive or negative regulatory connections among them (Blencowe et al., 2019; Haque et al., 2019). GRN can also be inferred from protein-protein interaction experiments (e.g., pull-down, yeast two-hybrid, or bimolecular fluorescence complementation) or from protein-DNA interaction experiments, such as yeast one-hybrid or ChIP-sequencing assays (de Matos Simoes et al., 2013). Particularly, GRN inferred from yeast one- and two-hybrid approaches have greatly contributed to our understanding of development and stress in Arabidopsis. These GRN have provided new insights into secondary cell wall gene regulation under abiotic stress (Taylor-Teeples et al., 2014), showed coordinated transcriptional regulation of enzymes involved in nitrogen metabolism (Gaudinier et al., 2018) and identified upstream regulation of AUXIN RESPONSE FACTORS to modulate auxin signaling throughout development (Truskina et al., 2020).

GRN inference algorithms have been classified into three major groups (Haque et al., 2019). The first group of methods uses linear and non-linear statistic correlation to measure the dependency between genes based on their expression patterns. These methods assume that the presence or absence of co-expressed transcripts reflects gene regulations. An improvement of this type of methods assumes that gene expression is deterministically controlled by upstream regulators. Based on this assumption, one of these methods, GENIST, first clusters putative regulated genes using gene expression data to subsequently model expression of each gene over time as a probabilistic function of itself and its putative upstream regulators, thus defining regulatory interactions (de Luis Balaguer et al., 2017). Secondly, probabilistic graphical models include other variables such as space. Thus, these methods are useful to reconstruct GRNs using samples collected from different cell types. At last, machine learning supervised and unsupervised methods have been used as an alternative to the previous methods. In the case of machine learning supervised methods, the algorithm is initially fed with previously demonstrated gene regulatory interactions (Haque et al., 2019). Machine learning analyses offer us algorithms not only for GRN inference but also for feature extraction across multi-dimensional datasets allowing integration of heterogeneous data from various high-throughput experimental techniques. As a result of GRN reconstruction, the relationships between genes can be established as direct or indirect (if one gene regulates another through an undefined intermediary) and signed (if the regulation determines activation –positive- or repression –negative of the downstream gene) or unsigned (if the type of regulation is unknown).

In plant biology, many GRN have been generated from RNA-seq experiments and these GRN have been proven to be useful to comprehend specific molecular processes (Haque et al., 2019). For example, a GRN predicting regulation of stem cells at the root apical meristem led to the identification of *TESMIN LIKE CXC2* as a master regulator of stem cell division (Clark et al., 2019). Similarly, the role of *PERIANTHIA* as regulator of the quiescent center was predicted by a GRN and further validated experimentally (de Luis Balaguer et al., 2017). GRN elucidated from RNA-seq experiments have also provided new insights into seed development (Ni et al., 2016).

GRN can also be generated from scRNA-seq data (Pratapa et al., 2020), which raises new challenges. For instance, GRN derived from scRNA-seq might be devoid of certain interactions related to the less abundant transcripts (as a consequence of lower depth of sequencing of scRNA-seq as compared with RNAseq). In contrast, GRN derived from scRNA-seq can identify TF-gene interactions at the single-cell level within a cell type or a tissue, therefore providing higher spatial resolution (Hu et al., 2020). Inferring GRN from scRNA-seq also represents a computational challenge as the transcriptomes of thousands of cells must be statistically analyzed and integrated to connect putative regulators (normally transcription factors) with downstream genes. Different methods to infer GRN from scRNA-seq have been developed (Pratapa et al., 2020). To improve reliability of the results, some methods such as GENIE3 initially feed the algorithm with specific information about the potential nodes or hubs (i.e., transcriptional regulators), which may regulate other genes (Huynh-Thu et al., 2010). Other methods such as SCODE or SINCERITIES require a time-course structure. In those cases, in which temporality of the dataset is not defined, pseudotime inference can be used to feed these methods with a relative time. Moreover, GENIE3, which reconstructs GRN from regression analyses of gene expression patterns using tree-based ensemble methods, also emerges as an alternative approach when temporal information is not available. Notably, GENIE3, has become one of the top performers when evaluated as benchmarking algorithm in DREAM4 (Marbach et al., 2012) and BEELINE (Pratapa et al., 2020). Furthermore Pratapa et al. (2020), shows that techniques that do not require pseudotime-ordered cells recover gene interactions more accurately.

Single-cell GRN inference methods have also been implemented to cope with problems intrinsic to scRNA-seq data, including those which are consequential to the so-called dropout effect. The dropout effect occurs when transcripts that are present in some cells show, however, zero reads in other cells; as this hampers the statistical analysis (Qiu, 2020). Moreover, scRNAseq data is affected by the variation in sequencing depth among cells and heterogeneity due to cell specialization or the cell cycle stage. Altogether, these issues affect GRN reconstruction from scRNA-seq and require specific methodology (Pratapa et al., 2020).

Once a GRN is generated from scRNA-seq data, analysis and mining of the network is greatly facilitated by software such as Cytoscape (Shannon, 2003). Cytoscape can be used to visualize and dissect the network as it can extract genes of interest and their neighbors, hubs (nodes highly connected), or filter specific relationships. In addition, Cytoscape integrates gene and pathway annotation, as well as expression patterns from external databases. The integration of this information into the network enables further analyses such as functional enrichment (based on gene ontology categories) or dissection of molecular pathways.

To facilitate a more profound understanding of the molecular processes related to development or stress, we propose that RNA-seq and GRN data are integrated with the new profiles and GRN obtained by scRNA-seq. However, many GRN were inferred for whole organs or sorted cells based on marker expression and lack single cell resolution. To address this issue, several deconvoluting methods can be used to infer (sub-)cell types or clusters of cells with specific transcriptomic signatures from tissues or bulk cells that have been sequenced by RNA-seq (Sun et al., 2019; Avila Cobos et al., 2020). scRNA-seq and deconvoluted RNA-seq data can then be systematically compared through the analysis of gene expression patterns, differentially expressed genes and reconstructed GRN using each dataset as input. Furthermore, scRNA-seq and deconvoluted RNA-seq datasets could be combined to reconstruct an integrated GRN. As an example of the potential of these approaches, a GRN reconstructed for trichoblast differentiation using scRNA-seq data and compared with a known GRN for root hair formation has further contributed to understanding this developmental process identifying new regulators (Denyer et al., 2019).

With the exception of the GRN reconstructed for trichoblast differentiation (Denyer et al., 2019), plant GRN do not normally integrate scRNA-seq data. Thus, current GRN do not consider developmental trajectories or intermediate transcriptomic states of cells, and thus this regulation has remained unexplored so far. With the use of scRNA-seq technology, intermediate transcriptomic states and cell trajectories can be integrated into GRN to gain further insight into the underlying molecular processes (Pratapa et al., 2020). GRN obtained through a cell lineage trajectory could increase the reliability of the network, as changes in gene relationships would be monitored with a higher resolution, which includes regulation of intermediate developmental stages. In this way, the molecular pathways regulating the developmental transitions or differentiation of the different cell linages of the primary root meristem could be more precisely defined.

### Experimental Validation

Single-cell omics analyses generate a huge amount of information such as cell trajectories, new types of cells (previously undetermined or misclassified), differentially expressed genes, and biomarkers. As these findings are typically based on statistical correlative analyses, they need to be experimentally assessed so their functional relevance can be determined.

A commonly used validation method to investigate expression patterns inferred from scRNA-seq analysis is to generate transcriptional reporters. In this approach, the promoter of a biomarker gene is transcriptionally fused to the *uidA* gene or a fluorescent protein coding sequence. Then, the reporter activity can be visualized under a (fluorescent) microscope to confirm expression of the biomarker gene in the cell type of interest (Reece-Hoyes and Walhout, 2018) or associated to a specific molecular process. Another option is performing fluorescence *in situ* hybridization (FISH) or colorimetric *in situ* hybridization (CISH) using the mRNA sequence of the biomarker as a probe (Femino et al., 1998; Marcino, 2013). Finally, functional validation of differentially expressed genes and cell type function can be investigated through loss-of-function mutants or overexpression lines (Capecchi, 1989; Visscher et al., 2015; Hahn et al., 2017).

The gene regulations established in a GRN can also be validated by perturbation experiments. These experiments are based on creating mutations in transcription factors or hubs of the network (for instance using the CRISPR/CAS9 technology). Subsequently, sc-RNAseq is performed in these mutants and the GRN is reconstructed again to test the edges and/or sign of the regulatory predictions of the initial GRN (Fiers et al., 2018).

## Unraveling the Heterogeneity and Temporality of Transcriptomic Changes

A major strength of scRNA-seq is the identification of scarce or new cell variants as well as of intermediate states of known cell types. The identification of these new types of cells suggests that formative or differentiation pathways are continuous dynamic processes, rather than a succession of homogeneous stages as previously profiled by microarrays or RNA-seq data using fluorescent markers that categorized cells into predefined cell types (Brady et al., 2007; Li et al., 2016).

The international consortium of the Human Cell Atlas Project aims to describe all the cell types in the human body in terms of their molecular signatures (Regev et al., 2017^2^). Contributions to this project have found new cell types in the different organs or tissues of the human body, e.g., retina (Lukowski et al., 2019), liver (Aizarani et al., 2019), or lungs (Braga et al., 2019). The generation of a Plant Cell Atlas Project has been proposed. The Plant Cell Atlas Project initiative will profile plants through scRNA-seq, proteomics and imaging, while all these datasets will be integrated using machine-learning algorithms. This initiative will likely accelerate discoveries in the field of plant science (Rhee et al., 2019).

Some scRNA-seq studies have been performed in plants, such as in the moss *Physcomitrella patens* (Kubo et al., 2019), maize (Nelms and Walbot, 2019; Satterlee et al., 2020; Bezrutczyk et al., 2021; Xu et al., 2021), rice (Liu et al., 2021) and the model plant *Arabidopsis thaliana* (Denyer et al., 2019; Jean-Baptiste et al., 2019; Ryu et al., 2019; Shulse et al., 2019; Zhang et al., 2019; Kim et al., 2021). The Arabidopsis root constitutes a model for stem cell and post-embryonic development. scRNA-seq of the Arabidopsis root has identified intermediate cellular states during cell differentiation. In these studies, not only cells from the main tissues were detected but also less-abundant cells such as the quiescent center and protoxylem cells. The information provided by these studies was thought to facilitate the future characterization of regulators involved in cell fate specification during root differentiation. As an initial approximation, matching pairs of transcriptional factors and their binding *cis* elements in the promoters of putatively downstream genes was carried out (Jean-Baptiste et al., 2019).

scRNA-seq studies in roots have also provided new insights into postembryonic development. Critical bifurcation points during cell differentiation have been identified by the use of pseudo-temporal trajectories (Jean-Baptiste et al., 2019). In addition, the sequential regulation of transcription factors to drive cell differentiation was proposed (Denyer et al., 2019). Detailed investigation of epidermal cells offers a good example of the possibilities of scRNA-seq techniques to comprehensively study cell differentiation. The trajectory from meristematic epidermal cells to fully differentiated root hair- or non-hair cells was traced. This approach resulted in the identification of an intermediate unknown identity for epidermal cells, which presented both hair- and non-hair-cell marker genes. This existence of this intermediate cell identity suggested that specification of epidermal cell fate would require a late decision in development. Further transcriptional information obtained from mutants impaired in specific types of epidermal cells identified the main regulators of epidermis differentiation and cell fate specification (Ryu et al., 2019). In addition, developmental trajectories of endodermis cells were investigated using scRNA-seq (Shulse et al., 2019).

scRNA-seq was used to study the regenerative capacity of root cells after excision of the root tip. After excision, the remaining cells undergo changes in cell identity that lead to the formation of a new functional meristem. Changes in cell identity during meristem regeneration are fast and organized. scRNA-seq studies showed the existence of predominant transitions in cell identity during the regeneration process, and identified the transcriptional changes associated with those transitions (Efroni et al., 2016). In agreement with the idea that some changes in cell identity are most likely to occur than others, ablation of single cells in roots specifically triggers the division of the adjacent cells on the external side. Subsequently, the daughter cells replace the damaged ones (Marhava et al., 2019). The use of scRNA-seq or other single-cell omics approaches could contribute to a better understanding of the regeneration processes of excised organs or ablated cells.

scRNA-seq not just limited to development or regeneration studies. Signaling and response to environmental changes may be interpreted differently by each cell. This hypothesis has been supported by scRNA-seq studies in the Arabidopsis root (Jean-Baptiste et al., 2019; Shulse et al., 2019). Although it is known that the heat response is not uniform across cells, scRNA-seq has shown that all cells belonging to the same cell type show unique and specific transcriptomic differences upon heat treatment (as compared with other cell types) (Jean-Baptiste et al., 2019). A cell-type specific response to heat is in agreement with previous research showing cell-type specific responses to other stresses (Dinneny et al., 2008), and demonstrates the versatility of single-cell approaches.

In addition to scRNA-seq techniques, other single-cell oriented studies or at cell resolution have been shown to be useful to unravel biological processes. For instance, analysis of the epigenetic state of single stomatal guard cells deciphered the specific role of H3K27me3 epigenetic mark during differentiation (Lee et al., 2019). Moreover, confocal laser microscopy techniques in roots allowed the investigation of biological processes with single-cell resolution (González-García et al., 2015; Long et al., 2017). A different example of a study performed with cell resolution was measuring the pace of the circadian clock in individual cells. This study concluded the existence of at least two main types of rhythms, one consisting of waves moving shootward and another moving rootward. This work shows a requirement for cell-to-cell communication in order to synchronize the clock and the subsequent outputs (Gould et al., 2018).

All these studies in plants demonstrate the existence of specific regulation in single cells. Therefore, a more extensive use of single cell-omics approaches could greatly contribute to a better understanding of the molecular processes taking place in individual cells, including how cells coordinate and facilitate functionality in a multicellular organism.

## Filling Gaps in Root Development: A Case for the Investigation of Lateral Root Formation

**FIGURE 2:**
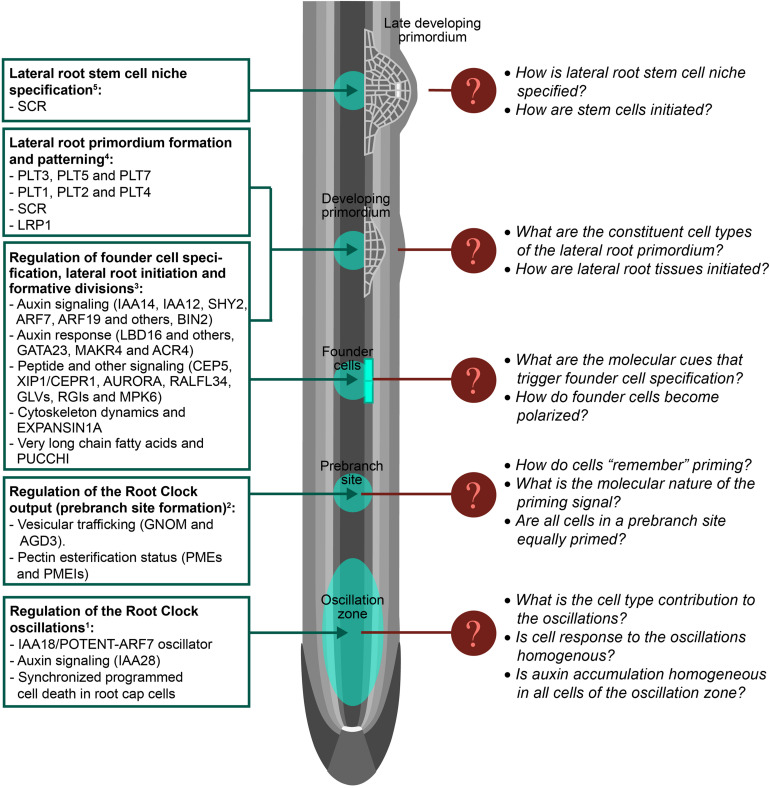
Filling the gaps in lateral root development. Schematic representation of an Arabidopsis primary root on which lateral root development stages are shown. Known regulation of lateral root formation is shown on the left. Note that regulators are indicated by abbreviated names. Unknown regulation or missing features of lateral root formation are indicated for specific developmental stages. Tissue layers are represented in gray. The quiescent center is represented in white: ^1^Please see references (De Smet et al., 2007; De Rybel et al., 2010; Moreno-Risueno et al., 2010; Xuan et al., 2015, 2016; Perianez-Rodriguez et al., 2021); ^2^(Wachsman et al., 2020); ^3^(Malamy and Benfey, 1997; Okushima et al., 2007; De Smet et al., 2008, 2010; De Rybel et al., 2010; Van Damme et al., 2011; Goh et al., 2012; Cho et al., 2013; Vermeer et al., 2014; Lavenus et al., 2015; Voß et al., 2015; Xuan et al., 2015; Murphy et al., 2016; Roberts et al., 2016; Ramakrishna et al., 2019; Trinh et al., 2019; Vilches Barro et al., 2019; Fernandez et al., 2020; Singh et al., 2020); ^4^(Malamy and Benfey, 1997; Goh et al., 2016; Du and Scheres, 2017; Singh et al., 2020); ^5^(Goh et al., 2016).

Although RNA-seq of sorted cell types, derived GRN and scRNA-seq have been used to study plant development, lateral root formation remains largely unexplored by these approaches (Lavenus et al., 2015; Voß et al., 2015). Lateral roots appear as repeated units along the primary root axis, however formation of lateral roots involves various pre-patterning and developmental stages (Malamy and Benfey, 1997; Lavenus et al., 2013). We will revise these developmental stages discussing how single-cell omics approaches might contribute to their molecular dissection (Figure 2).

### Lateral Root Pre-patterning Is Mediated by the Root Clock

Although lateral root formation is a plastic developmental process, the locations (prebranch sites) where these new organs form are defined by a time-dependent mechanism known as the Root Clock. The Root Clock was identified using the synthetic auxin-response promoter *DR5:Luciferase*, which rhythmically pulses in a region of the root tip known as the Oscillation Zone (OZ). Further transcriptomic analyses of this region showed changes in the expression of thousands of genes that fluctuated in or out of phase with DR5 (De Smet et al., 2007; Moreno-Risueno et al., 2010). It was later observed that auxin accumulation in the epidermis following programmed cell death of the root cap, as well as auxin signaling throughout the OZ contributed to the Root Clock pulses and affected subsequent prebranch site formation (De Rybel et al., 2010; Xuan et al., 2015, 2016). However, if auxin accumulates in the internal tissues of the OZ (other than the epidermis) is unresolved. The core oscillator of the Root Clock has been recently identified, demonstrating that negative auxin signaling regulation is critical for the Root Clock oscillations and establishes the periodicity of the system (Perianez-Rodriguez et al., 2021). In addition it was shown that the Root Clock oscillator can be entrained by external cues that lead to the periodic accumulation of auxin in the OZ such as during the gravitropic response. Even though the OZ is characterized by activity of the *DR5:Luciferase* marker, it remains unclear if the OZ can be understood as a homogeneous region with similar responses in all its constituent cells. Time-course scRNA-seq of the OZ might unravel the contribution of the different cell types to the oscillations, the molecular bases of cell synchrony during an oscillation, and if cell responses in the OZ are homogenous.

### Pre-branch Site Formation Involves an Unknown Cell Memory Mechanism

During root growth, new cells exit the meristem as they enlarge and differentiate. Thus the root longitudinal axis can be understood as developmental time: the older and more differentiated a cell is, the further away it will be from the meristem (Fisher and Sozzani, 2016; Perez-Garcia and Moreno-Risueno, 2018). Although all cells move across the OZ during root growth, only cells exposed to the peak of the in-phase oscillations become prebranch sites and show permanent activity of *DR5:Luciferase* (Moreno-Risueno et al., 2010; Xuan et al., 2015, 2016). Due to the dynamism of the Root Clock oscillations, cells enter and exit the OZ at different stages of the oscillations. This generated the hypothesis of whether cells get primed and are specified as prebranch sites depending on the phase of the oscillation (Traas and Vernoux, 2010). This hypothesis is in agreement with multilevel computational simulations of prebranch priming in the OZ, which shows that only reduced clusters of cells are exposed to maxima of the in-phase oscillations when they leave the OZ (Perianez-Rodriguez et al., 2021). Even though vesicular trafficking and cell wall remodeling affecting pectin esterification status have been shown to mediate Root Clock function leading to prebranch site specification (Wachsman et al., 2020) the molecular nature of the priming signal and its subsequent memorization by cells remains unresolved. Detailed single-cell omics studies of the OZ might help to understand cell memory and thus the molecular mechanism leading to prebranch site specification.

### Founder Cell Specification and Polarization Cues Have Not Been Identified

Primed xylem pole pericycle (XPP) cells, i.e., those in prebranch sites, are specified as lateral root founder cells (FC) in the differentiation zone. FC are cells which are able to initiate lateral root organogenesis, thus FC specification involves the acquisition of pluripotency. Next in the lateral root formation process, the nuclei of two adjacent FC migrate toward each other and FC divide asymmetrically to generate two morphological and presumably functionally different daughter cells. To date, the signal that triggers the specification of XPP cells into pluripotent FC is unknown. A number of regulators and molecular processes have been described to be part of this process and/or regulate FC division leading to lateral root initiation (Okushima et al., 2007; De Smet et al., 2008, 2010; De Rybel et al., 2010; Van Damme et al., 2011; Goh et al., 2012; Cho et al., 2013; Vermeer et al., 2014; Xuan et al., 2015; Murphy et al., 2016; Roberts et al., 2016; Ramakrishna et al., 2019; Trinh et al., 2019; Vilches Barro et al., 2019; Fernandez et al., 2020; Singh et al., 2020). In addition, several of these regulators have been shown to control subsequent formative divisions. As mutants for these regulators still have FC and their expression is not restricted to FC (Motte et al., 2019), it is unlikely that these regulators are the determinants of FC specification. The use of scRNA-seq in cells marked as prebranch sites and/or FC using available fluorescent reporters (Wachsman et al., 2020; Perianez-Rodriguez et al., 2021) could lead to unravel the molecular cues or determinants of FC specification. In addition, the signal that triggers FC polarization (if other than auxin) as well as the subsequent signaling cascade is unknown. The study of FC transcriptomes or proteomes with single-cell resolution could facilitate the identification of this putative signal and the subsequent polarization mechanism.

### Lateral Root Formation Requires Regulation of Cell Identity Transitions

After the first asymmetric division of FC, non-deterministic cell divisions take place to form the lateral root primordium (LRP) (De Smet et al., 2008; von Wangenheim et al., 2016). The tissues surrounding the LRP need to adapt to the new growing mass of cells causing opposing mechanical forces which play a role in determining the LRP shape (Lucas et al., 2013; Vermeer et al., 2014). However, the LRP is not a homogeneous mass of cells. A careful characterization of LRP formation in Arabidopsis has led to the classification of developmental stages which associate with specific marker expression and growth domains (Malamy and Benfey, 1997). These results suggest the early formation of tissues and specific regulation of cell fate in the LRP. More recently, it has been shown that meristem maintenance regulators of the primary root are expressed in specific subsets of cells of the LRP as well as their role in LRP patterning (Goh et al., 2016; Du and Scheres, 2017). These findings indicate the existence of distinctive cell identities in the LRP and a requirement for regulation of cell fate. A detailed single-cell transcriptional map during LRP formation and GRN reconstruction would reveal the ontogeny of the LRP, the constituent cell types or tissues, and how these would be initiated to eventually form a new lateral root.

### Establishment of a New Stem Cell Niche in the Lateral Root Primordium

PLETHORA (PLT) 3, PLT5, PLT7, and SCARECROW (SCR) factors are broadly expressed at the initial stages of LRP formation. Later on, PLT3, PLT5, and PLT7 activate PLT1, PLT2, and PLT4 in the central part of the LRP (Du and Scheres, 2017). SCR also shows enriched expression in the central part of the LRP after stage III/IV of development (Malamy and Benfey, 1997; Goh et al., 2016). This more restricted expression pattern of SCR, PLT1, PLT2, and PLT4 is coincident with activation of the quiescent center regulator WUSCHEL RELATED HOMEOBOX 5 (WOX5) and the establishment of an auxin maximum (Goh et al., 2016; Du and Scheres, 2017). Intriguingly, this process resembles regeneration of the primary root stem cell niche. Following quiescent center ablation, the combined action of the primary root meristem maintenance regulators (PLT1, PLT2, SCR, and SHORT-ROOT-SHR) leads to the confined expression of WOX5 and to the establishment of a new auxin maximum, which associates with stem cell niche re-specification (Xu et al., 2006). Resection of the root meristem leads to an embryo-like program of development in which expression of PLT1, PLT2, SCR, and SHR is re-organized preceding stem cell niche specification (Sena et al., 2009; Efroni et al., 2016). Given the similarities of these regenerative processes with lateral root formation, it is tempting to speculate that similar developmental mechanisms might exist. The use of scRNA-seq followed by the reconstruction of GRN might shed light into regulation of the developmental transitions leading to the establishment of a new stem cell niche during lateral root formation.

## Concluding Remarks

Single-cell omics technologies have been developed over the last few years, and more recently they have been implemented for plants. Notably, these technologies have facilitated the acquisition of results with unprecedented resolution for both animals and plants. The ability of the single-cell approaches (particularly of scRNA-Seq) to profile cell states has improved our understanding of cell functionality in multicellular organisms. With the use of scRNA-seq technology, new transcriptomic states and cell-types have been identified. Most of the new transcriptomic states have been interpreted as intermediate cell identities defining cell trajectories associated with development or differentiation. Single-cell datasets have also been used to identify gene regulatory interactions and different algorithms have been developed or implemented to generate GRN from scRNA-seq data. The integration of the new scRNA-seq and GRN with previous transcriptomic and GRN data has not been systematically explored, while such an approach could facilitate the identification of unknown regulatory mechanism. In addition, the integration of single-cell omics datasets with other heterogeneous data such as imaging or genetics (as proposed in the Plant Cell Atlas Project) could help to gain new insights into plant biology and development, likely contributing to unravel fundamental questions such as cell memory, synchronization, polarization, and pluripotency.

## Author Contributions

MM-R, LS-R, JC, and PP-G: conceptualization. LS-R, JC, and PP-G: methodology and writing-original draft. MM-R: resources, writing-review, editing, and supervision. MM-R and PP-G: funding acquisition. All authors contributed to the article and approved the submitted version.

## Conflict of Interest

The authors declare that the research was conducted in the absence of any commercial or financial relationships that could be construed as a potential conflict of interest.

## Footnotes

1 https://satijalab.org/

2 www.humancellatlas.org
